# Halo phenomenon on a congenital melanocytic nevus after dupilumab

**DOI:** 10.1016/j.jdcr.2025.05.045

**Published:** 2025-07-07

**Authors:** José Cuesta Camuñas, Iancarlos Jiménez Sacarello, Francisco Colón Fontánez

**Affiliations:** a Department of Dermatology, University of Puerto Rico School of Medicine, San Juan, Puerto Rico; b School of Medicine, University of Puerto Rico, Medical Sciences Campus, San Juan, Puerto Rico

**Keywords:** congenital melanocytic nevus, dupilumab, halo nevi

## Introduction

Halo nevus (HN), also known as leukoderma acquisitum centrifugum, is described as an acquired rim of hypomelanosis developing most commonly around a benign melanocytic nevus.^1^ It is hypothesized that the pathogenesis of HN stems from a humoral and cell-mediated immune response of the host to a nevus.^2^ Dupilumab, a fully human monoclonal antibody approved for the treatment of moderate-to-severe atopic dermatitis (AD), has recently been described to have several side effects, including induction of immune-mediated pigmentary disorders such as vitiligo.^3^ Here, we report the case of a patient with AD who developed an HN after starting dupilumab therapy. To our knowledge, there are no previously published reports describing an HN phenomenon potentially associated with dupilumab use.

## Case report

**Fig 1:**
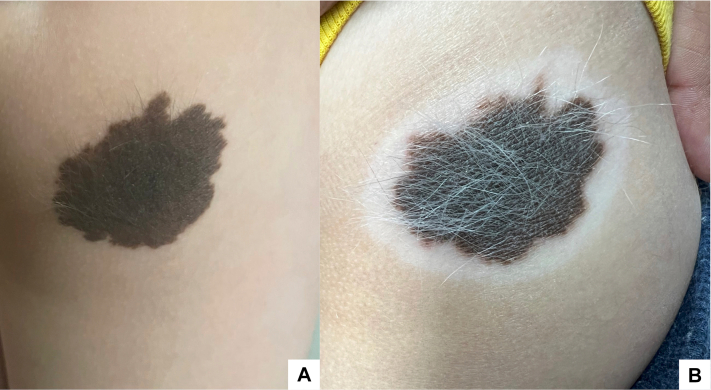
**A,** Congenital melanocytic nevus prior to development of halo phenomenon. **B,** 4.5 cm pigmented patch with *central white* hairs surrounded by hypopigmented halo on right buttock. The CMN pigment can also be seen disappearing at its edges. *CMN*, Congenital melanocytic nevus.

A 6-year-old male with past medical history of moderate AD, recently started on dupilumab therapy, was brought to a follow-up appointment due to a congenital melanocytic nevus (CMN) (Fig 1, *A*) that developed a depigmented halo around it. His mother noticed the hypopigmentation almost 1 year after starting dupilumab therapy. There was also associated whitening of the hairs on the CMN that was first noticed with the appearance of the halo nevi. On examination, a 4.5 × 2.5 cm dark brown patch was observed on the right buttock, surrounded by a broad depigmented halo and loss of pigmentation at the edges (Fig 1, *B*). Wood’s lamp examination showed no further areas of depigmentation in his body. No biopsy was performed considering that the findings were typical for an HN phenomenon.

## Discussion

Halo nevi are a rare dermatologic phenomenon characterized by a depigmented halo surrounding a melanocytic nevus.^1^ While they are generally considered benign, they are particularly uncommon when associated with CMN, with only few cases documented in the literature.^4^^,^^5^ An HN typically evolves as a depigmented ring around a pre-existing pigmented nevus over days to weeks. The central nevus may remain unchanged, lighten, or regress completely, leaving behind a depigmented patch of skin.^6^ This case is unique in that the HN developed approximately 1 year after the initiation of dupilumab therapy, raising the possibility of a causal relationship between the medication and the appearance of the halo.

Dupilumab, an interleukin-4 receptor alpha antagonist, works by inhibiting signaling pathways involved in the T helper type 2 (Th2) inflammatory response, which is predominant in conditions such as AD.^7^ This suppression of the Th2 response may lead to a relative increase in the T helper type 1 (Th1) immune pathway, suggested by the new onset or worsening of Th1/T helper 17–mediated conditions.^8^ Although the pathogenesis of halo nevi phenomena is not fully understood, it has been theoretically linked with an immune-mediated response, where CD8+ cytotoxic T cells, antigen presenting cells, and autoantibodies play a key role in melanocyte destruction and depigmentation.^2^^,^^9^ Given that dupilumab shifts the immune response from a Th2-dominant to a Th1-dominant state, it is plausible that this alteration could trigger similar immune mechanisms, promoting the development of halo nevi. Previous reports of dupilumab-associated vitiligo further support this potential immune shift as a contributing factor to this phenomenon.^3^ There have been 8 cases of dupilumab-associated vitiligo described in the english written medical literature.^3^^,^^10^ The immune modulation by dupilumab could potentially enhance cytotoxic T cell activity, leading to the depigmentation seen in these nevi, paralleling responses seen in vitiligo.

In conclusion, this case emphasizes the need for awareness of the rare dermatologic finding of halo nevi phenomena potentially associated with dupilumab therapy. While the appearance of these lesions can raise concern among patients and clinicians, reassurance and noninvasive management are often sufficient.^5^ This case may reflect an immune-mediated mechanism similar to that proposed in dupilumab-associated vitiligo, contributing to the development of the HN around a CMN. Further studies are needed to elucidate the mechanisms underlying this association, particularly the interplay between Th1 and Th2 immune responses, and to better understand the broader implications of immune modulation on melanocyte activity.

## Conflicts of interest

None disclosed.
